# Clinical Applicability of the Sellar Barrier Concept in Patients with Pituitary Apoplexy: Is It Possible?

**DOI:** 10.3390/life13010158

**Published:** 2023-01-05

**Authors:** José Ignacio Pailler, Juan Francisco Villalonga, Tomás Ries-Centeno, Amparo Saenz, Matías Baldoncini, Derek Orlando Pipolo, Eugenio Cárdenas Ruiz-Valdepeñas, Ariel Kaen, Lena Hirtler, David Roytowski, Domenico Solari, Andrés Cervio, Alvaro Campero

**Affiliations:** 1 LINT, Facultad de Medicina, Universidad Nacional de Tucumán, San Miguel de Tucumán 4000, Argentina; 2 Departamento de Neurocirugía, FLENI, Buenos Aires 1625, Argentina; 3 Hospital Virgen del Rocío, 41013 Sevilla, Spain; 4 Endoscopic Laboratory of Anatomy Center, Medical University of Vienna, 1090 Vienna, Austria; 5 Department of Neurosurgery, University of Cape Town, Cape Town 7701, South Africa; 6 Division of Neurosurgery, Department of Neurosciences, Reproductive and Odontostomatological Sciences, Universita’ degli Studi di Napoli Federico II, 80131 Naples, Italy

**Keywords:** sellar barrier, CSF leakage, skull base, pituitary apoplexy

## Abstract

There is evidence of association between sellar barrier thickness and intraoperative cerebrospinal fluid (CSF) leakage, impacting the postoperative prognosis of the patients. The aim of this study is to analyze the clinical applicability of the sellar barrier concept in a series of operated patients with pituitary apoplexy (PA). A retrospective study was conducted including 47 patients diagnosed with PA who underwent surgical treatment through a transsphenoidal approach. Brain magnetic resonance imaging (MRI) of the patients were evaluated and classified utilizing the following criteria: strong barrier (greater than 1 mm), weak barrier (less than 1 mm), and mixed barrier (less than 1 mm in one area and greater than 1 mm in another). The association between sellar barrier types and CSF leakage was analyzed, both pre- and intraoperatively. The preoperative MRI classification identified 10 (21.28%) patients presenting a weak sellar barrier, 20 patients (42.55%) with a mixed sellar barrier, and 17 patients (36.17%) exhibiting a strong sellar barrier. Preoperative weak and strong sellar barrier subtypes were associated with weak (*p* ≤ 0.001) and strong (*p* = 0.009) intraoperative sellar barriers, respectively. Strong intraoperative sellar barrier subtypes reduced the odds of CSF leakage by 86% (*p* = 0.01). A correlation between preoperative imaging and intraoperative findings in the setting of pituitary apoplexy has been observed.

## 1. Introduction

Cerebrospinal fluid (CSF) leakage is a major complication of transsphenoidal surgery (TSS), regardless of the technique implemented (microscopic, endoscopic assisted, or purely endoscopic) [1,2,3,4].

The limit between a pituitary tumor and suprasellar CSF is composed of three layers: arachnoid, identified as only constant tissue, dura mater of the sellar diaphragm, and the pituitary gland [5,6,7].

There is evidence of association between sellar barrier thickness and intraoperative cerebrospinal fluid (CSF) leakage, impacting the postoperative prognosis of the patient [5].

The sellar barrier concept was described by our team in 2019, with further sub-classifications developed and endoscopic anatomical concepts reinforced posteriorly [5,6,7]. Clinical applicability of this concept has been demonstrated in a multicenter prospective study and in a retrospective study, with specific reference to the elderly population [8,9]. However, the sellar barrier group has considered certain limitations to its applicability in various situations [9].

The aim of this study is to analyze the clinical applicability of the sellar barrier concept in a series of operated patients with pituitary apoplexy (PA).

## 2. Materials and Methods

A retrospective study was conducted including 47 patients who underwent TSS for PA. 

Exclusion criteria included: prior TSS, prior stereotactic radiosurgery, preoperative magnetic resonance imaging (MRI) lacking a specific protocol, absence of intraoperative video, and inadequate follow up. 

The procedures were carried out by surgeons experienced in pituitary surgery from five different institutions (LINT, FLENI, Seville, Naples, and Cape Town). The study was approved by the ethics committees of each institution. Every patient consented to the use of images and clinical data for the purpose of this research. Implementation and reporting of the study are in accordance with the STROBE guidelines (http://www.strobe-statement.org, accessed on 15 January 2020).

The demographic characteristics, sellar barrier type (radiological and intraoperative), and presence of CSF leakage of each patient were analyzed.

MRI scans were evaluated by neurosurgeons from each institution specializing in pituitary surgery. Two-dimensional intraoperative videos were analyzed by a second neurosurgeon from each institution for the assessment of barrier subtype and presence of CSF leakage. 

A specific MRI protocol was employed for optimal visualization of the sellar region: sagittal and coronal T1 and T2-weighted volumetric sequences, FLAIR and Echo Spin gradient sequences (1.5 T and 3.0 T), and contrast-enhanced T1-weighted volumetric sequences.

Evaluation of the sellar barrier was performed in a coronal projection at the level of the pituitary stalk and in a sagittal projection for determination of anteroposterior extension. Thin T2 slices were utilized for corroboration of contrast-enhanced sequences. 

The sellar barrier was measured in T1-weighted volumetric sequences using Kodak Carestream PACS-Client Suite v10 software. 

The barrier was considered strong when its thickness was greater than 1 mm, weak when less than 1 mm, and mixed when less than 1 mm in one area and greater than 1 mm in another.

### Statistical Analysis

Categorical variables were presented as absolute frequencies and percentages, while continuous variables were presented as mean and standard deviation. The association between pre- and intraoperative sellar barrier types was performed utilizing Chi^2^ and Fisher tests. A case-control odds ratio analysis was utilized for evaluating sellar barrier subtype and CSF leakage. Patients presenting CSF leaks were classified as cases and those without CSF leakage as controls. The odds ratios, with their respective 95% confidence intervals and *p*-values, were presented. The association between CSF leakage and the three types of sellar barriers was analyzed both pre- and intraoperatively. Significance was set at *p* < 0.05. STATA MP 14 (StataCorp, 4905 Lakeway Dr College Station, TX, USA) software was utilized for statistical analysis of the results.

## 3. Results

Of the original 223 patients within our database, 47 presented PA. Of these 47 patients, 29 were male (61.70%), and the mean age was 52.38 years.

Preoperative MRI classification of sellar barrier subtype displayed 10 (21.28%) patients presenting a weak sellar barrier, 20 patients (42.55%) with a mixed sellar barrier, and 17 patients (36.17%) showing a strong sellar barrier. 

**Figure 1:**
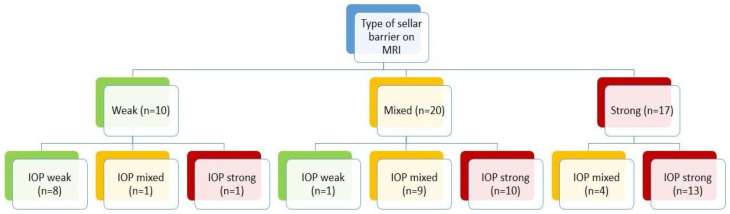
Results.

**Figure 2:**
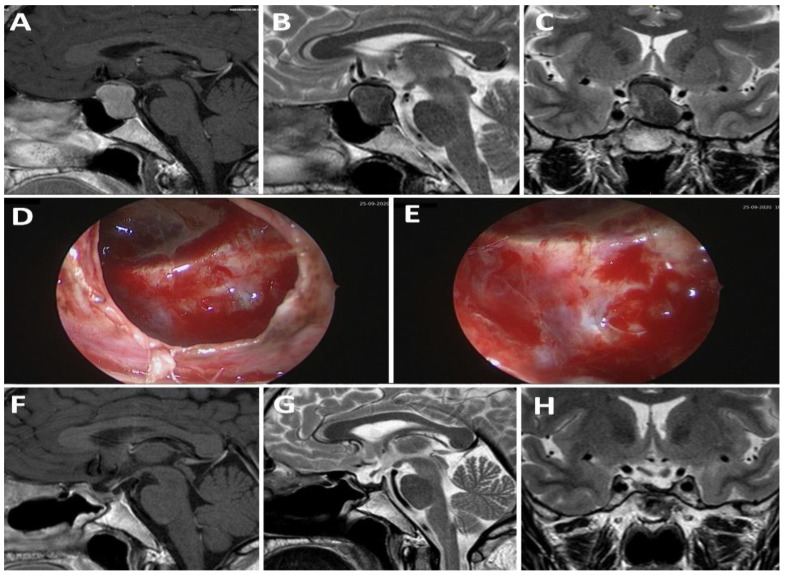
A 43-year-old patient presenting pituitary apoplexy in the context of a non-functioning macroadenoma. (**A**–**C**): Preoperative MRI; (**D**,**E**): intraoperative findings; (**F**–**H**): postoperative MRI.

**Figure 3:**
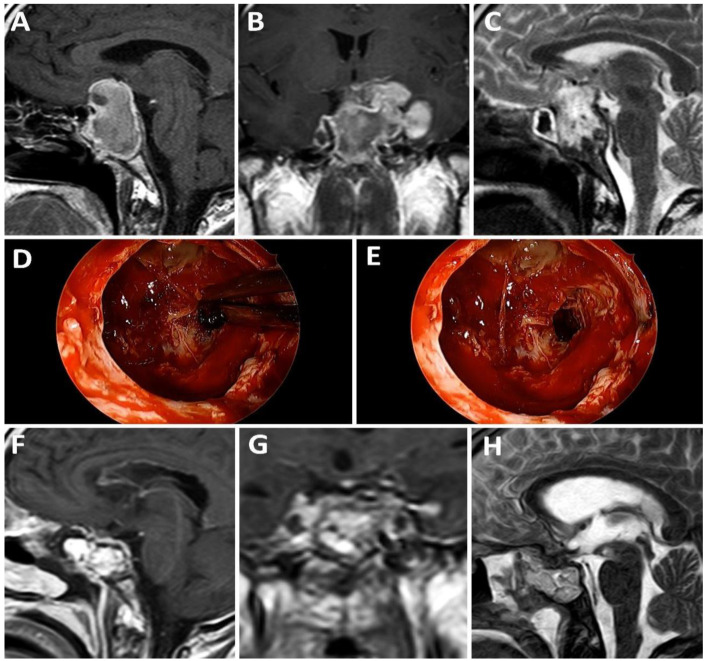
A 23-year-old patient with pituitary apoplexy in the context of a giant prolactinoma. (**A**–**C**): Preoperative MRI; (**D**,**E**): intraoperative findings; (**F**–**H**): postoperative MRI.

**Figure 4:**
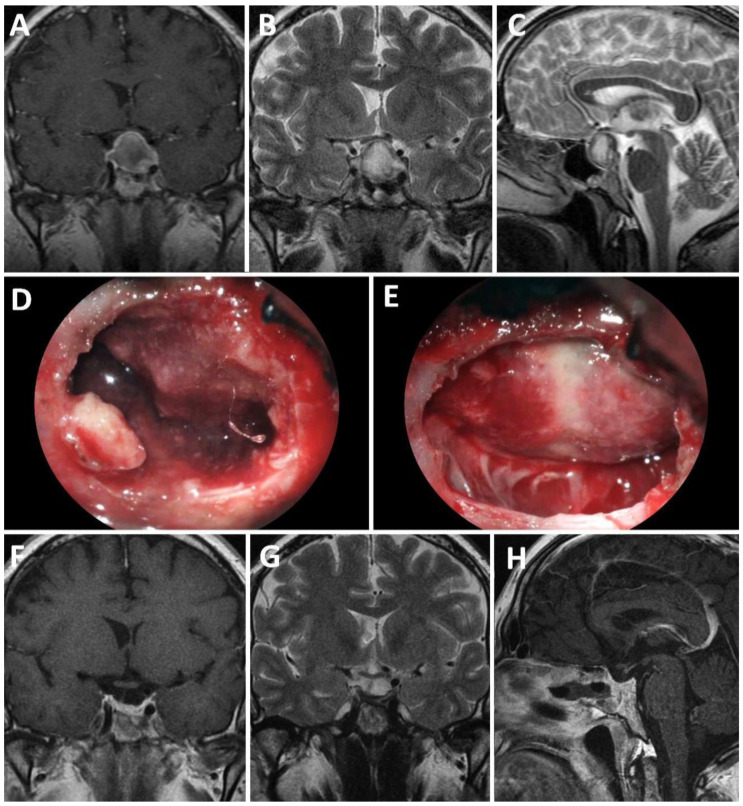
A 35-year-old patient with pituitary apoplexy in the context of a non-functioning macroadenoma. (**A**–**C**): Preoperative MRI; (**D**,**E**): intraoperative findings; (**F**–**H**): postoperative MRI.

Of the 10 patients with a weak sellar barrier in the preoperative MRI, 8 presented an intraoperatively confirmed weak barrier, 1 showed a mixed barrier, and 1 exhibited a strong barrier. A total of 20 patients presented a mixed sellar barrier according to MRI results. Of these, 9 were confirmed intraoperatively as mixed, 10 were confirmed as strong, and 1 was confirmed as weak. A total of 17 patients were classified as presenting a strong sellar subtype preoperatively; however, only 13 of these were confirmed intraoperatively as strong, while 4 presented a mixed subtype (Figure 1). Figure 2, Figure 3 and Figure 4 show representative cases of this series (Figure 2, Figure 3 and Figure 4).

A weak sellar barrier, identified on preoperative MRI, was associated with a weak sellar barrier during surgery (*p* ≤ 0.001). Pre- and intraoperative association was also observed for the strong sellar barrier subtype (*p* = 0.009). The mixed sellar barrier on MRI was not associated with a mixed sellar barrier during surgery (*p* = 0.051).

When analyzing the association between sellar barrier type and the presence of CSF leakage, a strong intraoperative sellar barrier reduced the odds of CSF leakage by 86% (OR = 0.14; CI95% 0.01–0.86; *p* = 0.01). None of the other types of sellar barriers were associated with this outcome variable. 

## 4. Discussion

### 4.1. Origin and Development of the Sellar Barrier Concept

The sellar barrier concept was created to emphasize the importance of the structures forming the roof of the pituitary fossa and to assess the risk of developing an intraoperative CSF fistula [5].

We initially identified and described two types of sellar barriers: strong and weak. Both were recognized on preoperative MRI and within the operative field. The association between the barrier types and the risk of developing a CSF fistula was demonstrated. The presence of a weak barrier was associated with a significantly higher risk of CSF leakage compared to the presence of a strong barrier [5]. The mixed sellar barrier was posteriorly described as the third type, sharing characteristics of both the strong and weak barriers [6].

To initially develop the sellar barrier concept, previous microsurgical anatomical studies of the pituitary fossa were utilized [10,11]. Considering the endonasal endoscopic approach as an effective technique for the treatment of pituitary tumors [1,3,12,13], we examined the microsurgical anatomy of the sellar barrier from an endonasal endoscopic point of view and confirmed its relevance in the clinical setting [7].

In the current phase of development of this concept, we seek to clarify when it is possible to identify the type of sellar barrier and its applicability for various pituitary pathologies.

### 4.2. Review of the Concept of Sellar Barrier [7,14]

#### 4.2.1. Prior Knowledge of Anatomy

In order to comprehend the components of the sellar barrier, we must first acknowledge the anatomical constitution of the roof of the pituitary fossa. 

The roof of the pituitary fossa is composed of the sellar diaphragm (dura mater) and the suprasellar arachnoid. The dura mater of the diaphragm is an extension of the dura mater of the roof of both cavernous sinuses [10]. 

The roof of the pituitary fossa, and particularly the opening of the sellar diaphragm, are highly variable, ranging from widely open, intermediately open, or closed [11]. 

#### 4.2.2. Definition of the Sellar Barrier

The sellar barrier is defined as an important limit between the pituitary tumor and the CSF, and is composed of up to three anatomical layers: arachnoid (the only portion which is constant), dura mater of the sellar diaphragm, and pituitary glandular tissue [5]. A structurally complete barrier is necessary to prevent CSF leakage.

The sellar barrier concept is best understood in a pathological scenario, such as when the tumor displaces the gland upward or laterally and adopts the morphology of the barrier [7]. 

#### 4.2.3. Identification of the Sellar Barrier with Imaging

MRI is the gold standard for identifying the sellar barrier. A specific sellar barrier MRI protocol must include sagittal and coronal slices in T1-weighted volumetric sequences, with and without contrast, as well as axial and sagittal slices of the sellar region in T2-weighted, FLAIR, and Echo Spin gradient sequences (with different 1.5 and 3.0 Tesla) [5]. T1-weighted volumetric sequences were utilized for measuring the sellar barrier using specific software (e.g., Horos v3.2.1 for Macintosh or Kodak Carestream PACS—Client Suite v10).

#### 4.2.4. Subtypes of Sellar Barriers 

Based on anatomical structure, two types of barriers were initially described: strong and weak [5]. The barrier was considered strong if it was composed of dura and/or the gland and weak if was constituted only by the arachnoid [5].

The addition of a mixed subtype to the classification was later proposed and defined as a combination of both previous types, partly strong and partly weak, which was validated in a series of operated patients [6].

In summary, there are currently three well-defined subtypes: strong, weak, and mixed.

#### 4.2.5. Differentiation Sellar Barrier Subtypes on MRI and during Surgery

Specific sellar region MRI protocols can identify sellar barrier subtypes. Measurements should be carried out with contrast-enhanced sequences. Barrier types are defined according to the following measurements [6]:

- Strong: barrier thickness more than 1 mm.
- Weak: barrier thickness less than 1 mm.
- Mixed: T1-weighted volumetric sequences displaying both types of thicknesses in different areas of the sellar barrier.

The three sellar barrier types can be distinguished intraoperatively, utilizing either microscopic or endoscopic techniques [5,7]:

- Strong: gland and/or dura mater are observed on the roof of the pituitary fossa.
- Weak: roof composed only of arachnoid.
- Mixed: a portion of the roof is covered by gland or dura mater and another portion by arachnoid.

#### 4.2.6. Correlation between MRI, Intraoperative Findings, and Risk of CSF Leakage

Currently published case series indicate that there is a strong correlation between radiological and intraoperative findings [5,6,7,8]: this correlation is reported to be up to 100% for weak barrier subtypes [6].

Patients with a weak sellar barrier present an increased risk of intraoperative CSF leakage. The strong sellar barrier is a protective factor for intraoperative CSF leakage [5,6,7,8].

#### 4.2.7. Usefulness of the Sellar Barrier Concept in Clinical–Surgical Practice 

The identification of a patient’s sellar barrier subtype is advantageous for clinical–surgical practice and for the physician–patient relationship. 

The application of this concept in clinical practice allows us to identify patients with a higher likelihood of requiring an aggressive reconstruction strategy. A relative indication for harvesting a nasoseptal flap is the presence of a weak sellar barrier on MRI. The sellar barrier concept serves as an adjunct for determining surgical decisions and preventing possible complications [7]. 

The patient can be informed of possible complications, including a higher risk of intraoperative CSF leakage and the potential need for an additional incision in order to utilize abdominal or crural fat for repair, if a weak barrier is noted by MRI. This could additionally support the medical–legal implications of this decision [5].

The sellar barrier concept is not useful for determining the surgical approach, which should be selected based on the type of tumor, degree of invasion, and extension of the lesion, among other characteristics. 

It is also not useful to define the reconstructive phase. The definitive reconstructive technique is defined according to the intraoperative degree of CSF leakage in the Esposito–Kelly classification [11]. 

#### 4.2.8. Impact of New Technologies on the Identification of the Sellar Barrier

Analyzing the pituitary region with a 7.0 Tesla MRI specific approach and subsequent sellar barrier classification could expand the knowledge of this entity and could further help define the mixed sellar barrier subtype [15]. Furthermore, a recent study determined that high definition endoscopes can aid in identifying small elements (e.g., a branch of the upper pituitary artery) intraoperatively [16]. This technology may help to avoid damage to the arachnoid surface and therefore, CSF leakage. Finally, 3D endoscopes will be a vital resource for demonstrating the concept in its various scenarios. 

### 4.3. Imaging Features of Pituitary Apoplexy and the Importance of Neuroimaging Protocols

PA is an acute condition that can be hemorrhagic or ischemic in origin. In most cases, it is associated with a pituitary macroadenoma and has a hemorrhagic component. CT and MRI are the most common imaging methods utilized in cases of PA. 

CT is effective in displaying expansive lesions of the pituitary gland with enlargement of the sellar region [17,18,19]. The hemorrhagic component of most PA is reflected on imaging as patchy or confluent areas of hyperdensity within a pituitary lesion [17,18,19,20]. The CT may be normal, underestimated, or not pathognomonic, mainly in atypical presentations with non-hemorrhagic PA or in the absence of a pituitary adenoma [21]. Although CT scans can show a sellar mass in up to 80% of cases, it is diagnostic in only 21–28% of patients [19,22,23,24,25]. Due to its rapid availability, it continues to be useful in the acute clinical setting [22]. Although CT is useful for the exclusion of other diseases during an emergency, MRI is the method of choice when AP is suspected [18,20,21].

MRI allows for confirmation of the diagnosis in more than 90% of cases, as well as a more detailed evaluation of the surrounding anatomical structures, providing an earlier diagnosis compared to that of CT [18,19,22,23,24,26,27,28,29]. PA typically presents as an expanding mass in the pituitary region. The intensity of the lesion in T1-weighted images (T1WI) and T2-weighted images (T2WI) is variable, depending on the stage of the hemorrhage. A hyperintense image will be observed on T1 due to the presence of blood (which may not be evident in the first hours) and will be established progressively from the periphery to the center of the lesion, which is produced by the transformation of deoxyhemoglobin to methemoglobin [29,30]. Correspondingly, T2W sequences will show irregular hypointense areas toward the center of the mass [28]. Gadolinium-enhanced images display partial tumoral enhancement in the periphery, but do not provide additional information regarding hemorrhage [29]. However, in cases of mixed hemorrhage and infarction, there may be areas of low signal intensity that do not enhance [20,31,32]. T2*-weighted gradient echo MRI is the most sensitive technique for the detection of brain hemorrhage, particularly for identifying intratumoral hemorrhage in pituitary adenomas using various forms of hypointense images: “rim,” “mass,” “spot,” “diffuse” and combinations of each of these [33,34,35,36,37]. This method is useful for evaluating both recent and older intratumoral hemorrhages in pituitary macroadenomas [37]. In the case of non-hemorrhagic PA, low signal intensity is evident in both T1WI and T2WI, accompanied by a peripheral rim enhancement in the contrast images [38]. Diffusion-weighted images can identify infarction in these cases, in which an increase in signal intensity is observed in relation to normal gray and white matter [27]. Other described MRI signs present in PA are the thickening of the sphenoid sinus mucosa during the acute phase of PA [39], the presence of a fluid level in the mass in late subacute hemorrhages [17,20,29,31], and the spontaneous shrinkage of the mass after a few weeks of conservative treatment [40].

PA is associated with multiple anatomical changes of the pituitary gland and the adjacent structures, translating into variable radiological presentations and leading us to consider the infeasibility for preoperative identification of the sellar barrier. However, advances in neuroimaging techniques and the development of a specific protocol for the sellar region (see Materials and Methods section) allow us to correctly differentiate glandular tissue, dura mater, and blood, even in the context of PA. In cases of adenomas not associated with PA, a contrast sequence may be sufficient to identify the sellar barrier. However, in the case of PA, the various sequences of the specific protocol play a major role. In this way, blood can be differentiated from other fluids and tissues.

### 4.4. Limitations

This is a retrospective study with a small series of patients. The results of this study may not be extrapolated to all neurosurgery centers, since they were obtained in institutions specialized in pituitary surgery that have specific MRI protocols. 

## 5. Conclusions

In this small series of patients, a correlation between preoperative imaging and intraoperative findings in the setting of PA has been observed. In the context of PA, the sellar barrier concept can be applied when a specific neuroimaging protocol for the sellar region is utilized.

## Data Availability

Not applicable.

## References

[1] Jho H.D., Carrau R.L. (1997). Endoscopic endonasal transsphenoidal surgery: Experience with 50 patients. J. Neurosurg..

[2] Black P.M., Zervas N.T., Candia G.L. (1987). Incidence and management of complications of transsphenoidal operation for pituitary adenomas. Neurosurgery.

[3] Cappabianca P., Cavallo L.M., de Divitiis E. (2004). Endoscopic endonasal transsphenoidal surgery. Neurosurgery.

[4] Strickland B.A., Lucas J., Harris B., Kulubya E., Bakhsheshian J., Liu C., Wrobel B., Carmichael J.D., Weiss M., Zada G. (2018). Identification and repair of intraoperative cerebrospinal fluid leaks in endonasal transsphenoidal pituitary surgery: Surgical experience in a series of 1002 patients. J. Neurosurg..

[5] Campero A., Villalonga J.F., Basso A. (2019). Anatomical Risk Factors for Intraoperative Cerebrospinal Fluid Leaks During Transsphenoidal Surgery for Pituitary Adenomas. World Neurosurg..

[6] Villalonga J.F., Ries-Centeno T., Saenz A., Solari D., Cervio A., Campero A. (2019). The Mixed Sellar Barrier: A New Subtype of this Novel Concept. World Neurosurg..

[7] Villalonga J.F., Fuchssteiner C., Solari D., Campero A., Cavallo L.M., Cappabianca P., Hirtler L. (2020). Endoscopic anatomy of the sellar barrier: From the anatomical model to the operating room. Clin. Anat..

[8] Centeno T.R., Villalonga J.F., Saenz A., Del Pont F.M., Cervio A., Campero A. (2020). The sellar barrier and intraoperative CSF leak in elderly patients. J. Clin. Neurosci..

[9] Villalonga J.F., Solari D., Cavallo L.M., Cappabianca P., Prevedello D.M., Carrau R., Martinez-Perez R., Hardesty D., Fuchssteiner C., Saenz A. (2021). The sellar barrier on preoperative imaging predicts intraoperative cerebrospinal fluid leak: A prospective multicenter cohort study. Pituitary.

[10] Campero A., Campero A.A., Martins C., Yasuda A., Rhoton A.L. (2010). Surgical anatomy of the dural walls of the cavernous sinus. J. Clin. Neurosci..

[11] Campero A., Martins C., Yasuda A., Rhoton A.L. (2008). Microsurgical anatomy of the diaphragma sellae and its role in directing the pattern of growth of pituitary adenomas. Neurosurgery.

[12] Carrau R.L., Jho H.D., Ko Y. (1996). Transnasal-transsphenoidal endoscopic surgery of the pituitary gland. Laryngoscope.

[13] Cappabianca P., Alfieri A., Thermes S., Buonamassa S., de Divitiis E. (1999). Instruments for endoscopic endonasal transsphenoidal surgery. Neurosurgery.

[14] Villalonga J.F., Martinez Font A., Campero A. (2020). The sellar barrier’s: An important new concept in pituitary surgery. Arch. Neurosurg..

[15] De Rotte A.A., van der Kolk A.G., Rutgers D., Zelissen P.M., Visser F., Luijten P.R., Hendrikse J. (2014). Feasibility of high-resolution pituitary MRI at 7.0 tesla. Eur. Radiol..

[16] Doglietto F., Prevedello D.M., Belotti F., Ferrari M., Lancini D., Schreiber A., Raffetti E., La Rocca G., Rigante M., Lauretti L. (2019). The Superior Hypophyseal Arteries: Anatomical Study with an Endoscopic Endonasal Perspective. Oper. Neurosurg..

[17] Dubuisson A.S., Beckers A., Stevenaert A. (2007). Classical pituitary tumour apoplexy: Clinical features, management and outcomes in a series of 24 patients. Clin. Neurol. Neurosurg..

[18] Randeva H.S., Schoebel J., Byrne J., Esiri M., Adams C.B., Wass J.A. (1999). Classical pituitary apoplexy: Clinical features, management and outcome. Clin. Endocrinol..

[19] Ayuk J., McGregor E.J., Mitchell R.D., Gittoes N.J. (2004). Acute management of pituitary apoplexy—Surgery or conservative management?. Clin. Endocrinol..

[20] Semple P.L., Jane J.A., Lopes M.B., Laws E.R. (2008). Pituitary apoplexy: Correlation between magnetic resonance imaging and histopathological results. J. Neurosurg..

[21] Boellis A., di Napoli A., Romano A., Bozzao A. (2014). Pituitary apoplexy: An update on clinical and imaging features. Insights Imaging.

[22] Rajasekaran S., Vanderpump M., Baldeweg S., Drake W., Reddy N., Lanyon M., Markey A., Plant G., Powell M., Sinha S. (2011). UK guidelines for the management of pituitary apoplexy. Clin. Endocrinol..

[23] Onesti S.T., Wisniewski T., Post K.D. (1990). Clinical versus subclinical pituitary apoplexy: Presentation, surgical management, and outcome in 21 patients. Neurosurgery.

[24] Sibal L., Ball S.G., Connolly V., James R.A., Kane P., Kelly W.F., Kendall-Taylor P., Mathias D., Perros P., Quinton R. (2004). Pituitary apoplexy: A review of clinical presentation, management and outcome in 45 cases. Pituitary.

[25] Kaplan B., Day A.L., Quisling R., Ballinger W. (1983). Hemorrhage into pituitary adenomas. Surg. Neurol..

[26] Bills D.C., Meyer F.B., Laws E.R., Davis D.H., Ebersold M.J., Scheithauer B.W., Ilstrup D.M., Abboud C.F. (1993). A retrospective analysis of pituitary apoplexy. Neurosurgery.

[27] Rogg J.M., Tung G.A., Anderson G., Cortez S. (2002). Pituitary apoplexy: Early detection with diffusion-weighted MR imaging. AJNR Am. J. Neuroradiol..

[28] Briet C., Salenave S., Bonneville J.F., Laws E.R., Chanson P. (2015). Pituitary Apoplexy. Endocr. Rev..

[29] Piotin M., Tampieri D., Rufenacht D.A., Mohr G., Garant M., Del Carpio R., Robert F., Delavelle J., Melanson D. (1999). The various MRI patterns of pituitary apoplexy. Eur. Radiol..

[30] Glick R.P., Tiesi J.A. (1990). Subacute pituitary apoplexy: Clinical and magnetic resonance imaging characteristics. Neurosurgery.

[31] Kurihara N., Takahashi S., Higano S., Ikeda H., Mugikura S., Singh L.N., Furuta S., Tamura H., Ishibashi T., Maruoka S. (1998). Hemorrhage in pituitary adenoma: Correlation of MR imaging with operative findings. Eur. Radiol..

[32] Lazaro C.M., Guo W.Y., Sami M., Hindmarsh T., Ericson K., Hulting A.L., Wersall J. (1994). Haemorrhagic pituitary tumours. Neuroradiology.

[33] Unger E.C., Cohen M.S., Brown T.R. (1989). Gradient-echo imaging of hemorrhage at 1.5 Tesla. Magn. Reson. Imaging.

[34] Alemany Ripoll M., Stenborg A., Sonninen P., Terent A., Raininko R. (2004). Detection and appearance of intraparenchymal haematomas of the brain at 1.5 T with spin-echo, FLAIR and GE sequences: Poor relationship to the age of the haematoma. Neuroradiology.

[35] Arnould M.C., Grandin C.B., Peeters A., Cosnard G., Duprez T.P. (2004). Comparison of CT and three MR sequences for detecting and categorizing early (48 h) hemorrhagic transformation in hyperacute ischemic stroke. AJNR Am. J. Neuroradiol..

[36] Kidwell C.S., Chalela J.A., Saver J.L., Starkman S., Hill M.D., Demchuk A.M., Butman J.A., Patronas N., Alger J.R., Latour L.L. (2004). Comparison of MRI and CT for detection of acute intracerebral hemorrhage. JAMA.

[37] Tosaka M., Sato N., Hirato J., Fujimaki H., Yamaguchi R., Kohga H., Hashimoto K., Yamada M., Mori M., Saito N. (2007). Assessment of hemorrhage in pituitary macroadenoma by T2*-weighted gradient-echo MR imaging. AJNR Am. J. Neuroradiol..

[38] Ostrov S.G., Quencer R.M., Hoffman J.C., Davis P.C., Hasso A.N., David N.J. (1989). Hemorrhage within pituitary adenomas: How often associated with pituitary apoplexy syndrome?. AJR Am. J. Roentgenol..

[39] Arita K., Kurisu K., Tominaga A., Sugiyama K., Ikawa F., Yoshioka H., Sumida M., Kanou Y., Yajin K., Ogawa R. (2001). Thickening of sphenoid sinus mucosa during the acute stage of pituitary apoplexy. J. Neurosurg..

[40] Armstrong M.R., Douek M., Schellinger D., Patronas N.J. (1991). Regression of pituitary macroadenoma after pituitary apoplexy: CT and MR studies. J. Comput. Assist. Tomogr..

